# *Trypanosoma cruzi* genetic diversity: impact on transmission cycles and Chagas disease

**DOI:** 10.1590/0074-02760210193

**Published:** 2022-05-06

**Authors:** Bianca Zingales, Daniella C Bartholomeu

**Affiliations:** 1 Universidade de São Paulo, Instituto de Química, Departamento de Bioquímica, São Paulo, SP, Brasil; 2 Universidade Federal de Minas Gerais, Instituto de Ciências Biológicas, Departamento de Parasitologia, Belo Horizonte, MG, Brasil

**Keywords:** discrete typing units, eco-epidemiology, geographical distribution, Chagas disease manifestations, genomic variability, multigene families

## Abstract

*Trypanosoma cruzi*, the agent of Chagas disease (ChD), exhibits remarkable biological and genetic diversity, along with eco-epidemiological complexity. In order to facilitate communication among researchers aiming at the characterisation of biological and epidemiological aspects of *T. cruzi*, parasite isolates and strains were partitioned into seven discrete typing units (DTUs), TcI-TcVI and TcBat, identifiable by reproducible genotyping protocols. Here we present the potential origin of the genetic diversity of *T. cruzi* and summarise knowledge about eco-epidemiological associations of DTUs with mammalian reservoirs and vectors. Circumstantial evidence of a connection between *T. cruzi* genotype and ChD manifestations is also discussed emphasising the role of the host’s immune response in clinical ChD progression. We describe genomic aspects of DTUs focusing on polymorphisms in multigene families encoding surface antigens that play essential functions for parasite survival both in the insect vector and the mammalian host. Such antigens most probably contributed to the parasite success in establishing infections in different hosts and exploring several niches. Gaps in the current knowledge and challenges for future research are pointed out.

*Trypanosoma cruzi* is genetically very diverse and the understanding of the origin of the diversity and population structure of this parasite is fundamental because of the connections to transmission cycles and Chagas disease (ChD) characteristics.

Chagas infection, initially a wild enzooty, maintained by sylvatic mammals and transmitted by hematophagous triatomine species, became an anthropozoonosis with the entry of man into the natural environment in which *T. cruzi* circulated. The occupation of these spaces by man caused some species of triatomines to be introduced, actively or passively, in human dwellings and new cycles of transmission were established. Thus, man and domestic animals became part of the epidemiological chain of ChD, with the possibility of exchanging *T. cruzi* between the sylvatic and domestic cycles.

In this scenario, it is inferred that a variety of mammalian hosts and vectors must have exerted different selective pressures over time, which resulted in a broad variety of parasite populations that have deep biological and genetic differences. The aim of this review is to draw attention to some aspects of *T. cruzi* genetic diversity and its impact on the eco-epidemiology and characteristics of ChD.

**Evolutionary history of *T. cruzi***

The first document of human infection by *T. cruzi* was obtained from mummies of 9,000 years of the Chinchorro civilisation that inhabited the coastal region of Chile and Peru.^1^ The presence of *T. cruzi* DNA in other mummies made it possible to estimate a prevalence rate for ChD of 40.6%.^1^ These findings suggested an active *T. cruzi* transmission process and a robust wild cycle that should have been well established when humans entered the region.

The exact date of *T. cruzi*’s emergence in the Americas is not known. Phylogenetic analyses of 18S rRNA gene sequences served as the basis for the southern super-continent hypothesis, according to which the ancestor of the genus *Trypanosoma* would be present in Pangea before the separation of the continents during the mesozoic era (~ 230 mya).^2^ From this ancestor, salivarian trypanosomes (the “*T. brucei* clade”, that includes the African trypanosomes) and stercorarian trypanosomes (the “*T. cruzi* clade”, that comprises *T. cruzi*, *T. rangel*i, various trypanosomes of bats, and a species from an Australian kangaroo) diverged approximately 100 mya. Following the continental split of Africa and South America, *T. brucei* emerged in the Old World and *T. cruzi* in the New World (revised by Steverding).^3^

Later, the southern super-continent trypanosome hypothesis has been challenged based on the analysis of several genetic markers of trypanosomes from bats of Africa, Old and New World.^4^ ^,^ ^5^ ^,^ ^6^ The new theory, known as the *bat seeding hypothesis*,^4^ proposes that the “*T. cruzi* clade” evolved within a clade of bat trypanosomes. According to this scenario, the common ancestor of *T. cruzi* was a bat trypanosome that switched from bats into terrestrial mammals in the New World. One of these switches originated *T. cruzi*. Since molecular phylogenies of New World bats suggest that trypanosome-infected bats have colonised South America about 7-10 mya,^7^ this date could indicate the arrival of *T. cruzi* in the Americas.

Schofield^8^ developed a brilliant theory to explain the evolution of the association between *T. cruzi* and its triatomine vectors. In this theory, the earliest forms of *T. cruzi* would be associated with marsupial opossums that would have arrived in South America after the separation of the continents about 40 mya. The transmission of *T. cruzi* between opossums would have occurred by the predation of infected animals or via their anal gland secretions.^9^ By the late tertiary or early pleistocene, some 2-5 mya, opossums would be common in South America, along with armadillos and cricetid rodents. In this period, reduvid predators would also be present and would feed on the blood of opossums infected with *T. cruzi* spreading trypanosomes to new hosts. Schofield^8^ proposed that, when passing through different mammalian and triatomine hosts, *T. cruzi* would have suffered different selective pressures that led to the appearance of new parasite “variants” that today are distinguishable by a series of genetic markers.

***Trypanosoma cruzi* population structure and discrete typing units (DTUs)**

Several studies, many reported in the book *Trypanosoma cruzi* and Chagas disease by Zigman Brener and Zilton Andrade,^10^ clearly illustrate the biological differences between *T. cruzi* populations recovered from various hosts. Morphological differences are evident, also documented in the drawings of Carlos Chagas in 1909,^11^ as well as variance of parasitaemia curves and mortality in murine models, therapeutic response to different drugs and tissue tropism. In the years that followed the book publication, it became evident that the phenotypic differences are the result of profound genotypic heterogeneity of *T. cruzi* isolates.

The population structure of a species may be defined by the organisation of genetic variation that is driven by the combined effects of evolutionary processes that include recombination, mutation, genetic drift, demographic history, and natural selection.^12^ The population structure of *T. cruzi* explains the genetic differences observed among the isolates (see below).

*T. cruzi* replicates by binary fission. For some time, *T. cruzi* was considered the paradigm for clonal evolution in parasitic protozoa, in which genetic exchange was rare or absent.^13^ However, experimental crosses and advancements in whole-genome sequencing have now proved that *T. cruzi* has capacity for genetic exchange, with potential to generate new genotypes.

*T. cruzi*’s genetic recombination was elegantly demonstrated in infected mammalian cells using parental parasites carrying different drug-resistance genes.^14^ Double-drug-resistant progenies were recovered, which were subtetraploid and had DNA content ~70% higher than the parental strains.^15^ It is surprising that after this date no additional evidence of *T. cruzi* recombination in other systems has been provided. Putative mechanisms for *T. cruzi* recombination have been proposed.^16^ At present it is accepted that genetic exchange has a strong impact on the current evolutionary and epidemiological characteristics of this parasite.

Besides understanding the origin of the biological differences among the isolates, researchers were challenged to explain why about 70% of individuals chronically infected with *T. cruzi* remained asymptomatic, while 20% to 30% developed cardiac manifestations (Chronic Chagas Cardiomyopathy, CCC) and 10% to 15% digestive syndromes (DIG).^17^

The first step in the study of the characteristics of *T. cruzi* isolates was to search for molecular markers that would cluster the isolates into discrete groups. Our laboratory in collaboration with researchers from Fiocruz, UFMG and UCLA showed that a 24Sα rRNA gene sequence, the intergenic region of the mini-exon gene and randomly amplified polymorphic DNA (RAPD) indicated the clear division of *T. cruzi* into two major lineages presenting a high phylogenetic divergence.^18^ In addition, our studies showed, for the first time, the existence of hybrid strains. Fig. 1 shows the ability of the 24Sα rRNA gene sequence and the mini-exon intergenic region to discriminate *T. cruzi* genotypes. The biochemical, epidemiological characteristics and possible models of evolution of the two lineages were discussed in a subsequent work.^19^

**Fig. 1:**
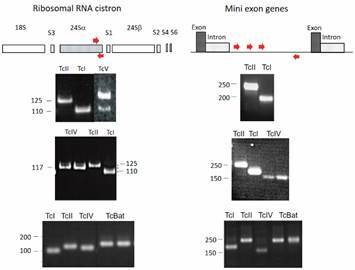
the 24Sα rRNA gene sequence and the intergenic region of the mini-exon gene are valuable markers to genotype *Trypanosoma cruzi* isolates. Schematic representation of *T. cruzi* Ribosomal RNA cistron and Mini exon genes. The arrows indicate the position of primers used for polymerase chain reaction (PCR) amplification of gene regions. The amplification products for different discrete typing units (DTUs) after gel electrophoresis are shown.

In a satellite meeting held at Fiocruz in 1999, commemorating the 90th anniversary of the discovery of ChD, an expert committee revised the available knowledge that indicated a convergence toward clustering *T. cruzi* strains into two major groups, and recommended that these groups should be named *T. cruzi* I and *T. cruzi* II. The classification of apparent hybrid strains would be decided later after further studies.^20^

The studies continued and in a second satellite meeting held in Buzios, in 2009, it was recognised that *T. cruzi* strains should be classified into six DTUs, TcI-TcVI.^21^ The term ‘‘discrete typing unit’’ was proposed to describe sets of stocks that are genetically more similar to each other than to any other stock and are identifiable by common molecular markers.^22^ It is important to note that in the new nomenclature TcI corresponds to the *T. cruzi* I group mentioned above^20^ and that *T. cruzi* II group was subdivided into four DTUs, TcII-TcVI. In addition, it was acknowledged that DTUs TcV and TcVI are hybrids. The rationale for the new nomenclature, epidemiological relevance and research applications have been discussed elsewhere.^23^ ^,^ ^24^

Several models have been presented for the evolution of hybrid DTUs. All the approaches agree that TcI and TcII are two pure lineages that are evolving separately for a long time and that TcV and TcVI have a hybrid origin with TcII and TcIII as putative parentals. The evolution of TcIII and TcIV has been a matter of intense debate and it is outside the scope of this article to discuss the proposed models. Relevant references can be found elsewhere.^23^ ^,^ ^24^

A seventh DTU, called TcBat, was discovered in some bat species in Brazil.^25^ PCR amplification of the region of the 24Sα rRNA gene of TcBat originated a product of ~ 140 bp, in contrast to that observed for the major *T. cruzi* I and II lineages^18^ (see Fig. 1). Subsequent studies based on multiple molecular markers support the proximity of TcBat to the DTU TcI.^26^ Besides Brazil, TcBat was reported in bats from other south American countries^27^ ^,^ ^28^ and TcBat DNA was disclosed in the heart of mummies from Chile^29^ and in a child in Colombia.^30^

Aiming at mapping the geographical distribution of DTUs and defining their potential association with mammalian hosts and vectors, genotyping protocols, that could be used by researchers from ChD endemic regions were proposed.^23^ For population genetic studies, the analysis of polymorphic microsatellites and multilocus sequence typing (MLST), based on DNA sequence variations in a number of genes, are recommended.^23^ Importantly, these approaches and whole genome sequencing revealed intra-DTU genetic variability.^31^ ^,^ ^32^ ^,^ ^33^

**Association of *Trypanosoma cruzi* DTUs with mammalian reservoirs and vectors**

*Mammalian reservoirs* - *T. cruzi* is capable of infecting more than 100 species of mammals: opossums, armadillos, bats, carnivores, rodents and primates, many of which are found in the sylvatic environment. There, the organisms are exposed to infection by different DTUs, once or multiple times, either orally, by predation of triatomines or other mammals, or by inoculation by triatomine hematophagous species. Oral transmission seems to be the most efficient process for the propagation of *T. cruzi* in nature and the molecular mechanisms of the infection by oral route have been unveiled by Nobuko Yoshida’s group (reviewed in^33^). Once infected, each mammalian species exerts different evolutionary pressures on the parasite, which determine the selection of sub-populations. As a result, each host species plays a different role in the complex transmission network of *T. cruzi* in distinct habitats and biomes. These mammalian species are called reservoir hosts, which, according to Ashford’s definition, guarantee the indefinite survival of the infectious agent in an ecological system.^34^

The presence of *T. cruzi* DTUs in mammalian reservoirs and vectors has been investigated using different genotyping protocols, with varying sensitivity and specificity, and molecular phylogenetic approaches.

Several articles and reviews describe the distribution of *T. cruzi* DTUs in wild reservoirs.^23^ ^,^ ^24^ ^,^ ^35^ ^,^ ^36^ ^,^ ^37^ ^,^ ^38^ In the comprehensive review by Jansen and coworkers, the complexity of *T. cruzi* sylvatic transmission cycle is discussed, as well as biological characteristics of the reservoirs and distribution of *T. cruzi* genotypes in Brazilian biomes.^37^ Here, due to space limitations, only the consensus of the preferential association among wild mammals and DTUs is summarised and is depicted in Fig. 2.

**Fig. 2:**
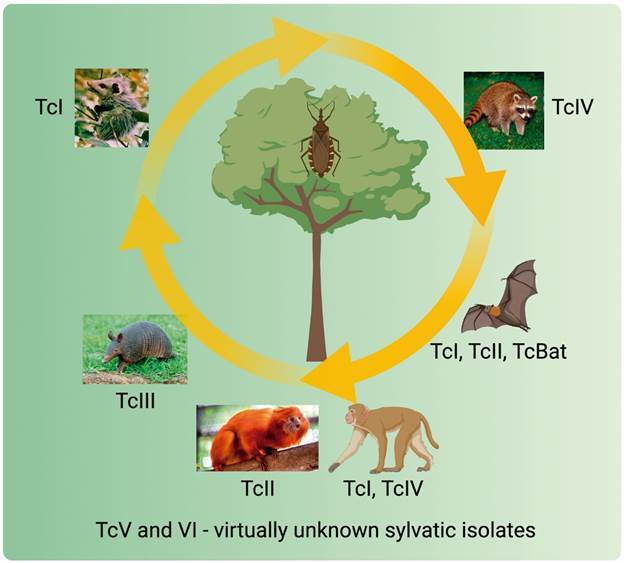
sylvatic reservoirs of *Trypanosoma cruzi* discrete typing units (DTUs). The figure illustrates the consensus of the preferential, but not exclusive, association of DTUs to certain species / orders of wild mammals. Created with Biorender.com.

*Opossums and DTU TcI* - It is indisputable that TcI is the major DTU found infecting *Didelphis* opossums. In pioneering studies, Maria Deane and co-workers showed that *D. marsupialis* displayed a very distinct infection pattern when inoculated with the Y strain (TcII) or with the F strain (TcI).^39^ While the F strain promoted blood-noticeable parasitaemia, the Y strain was not detected in the blood circulation. Thus, these observations suggest that at least some *Didelphis* species are able to control and even eliminate some populations of DTU TcII.

At this point, several questions can be raised. What happens to the other DTUs? Are they also eliminated by opossums? By what mechanisms does elimination occur? Does TcI DTU adapted to opossums have genetic characteristics that differentiate it from TcI DTUs found in other hosts? Answers to these questions are relevant not only from the academic point of view but also for the comprehension of host-parasite interactions and mechanisms involved in this adaptation.

*Armadillos and DTU TcIII* - Genotyping of *T. cruzi* isolates from armadillos captured in different regions indicate that these mammals are natural hosts of DTU TcIII.^35^ ^,^ ^40^ ^,^ ^41^ In an extensive eco-epidemiological study in the Paraguayan Chaco region, Yeo and collaborators verified the prevalence of TcIII in different species of armadillos and, to a lesser extent, mixed infections with TcIII, TcII and TcV.^35^ Since previous data indicated that DTU TcV was the result of the hybridisation of TcII and TcIII parentals, the authors proposed that armadillos, harboring co-infections, could be the intermediate host that facilitated the evolution of hybrid lineages. However, as discussed below, TcV is rare in the wild and predominates in domestic transmission cycles in Bolivia, Chile and Paraguay. Therefore, a topic to be clarified is the process by which TcV has established itself in the domestic environment.

*Bats and DTU TcBAT* - TcBAT was detected in bats captured in four Brazilian biomes and in Panama, Colombia and Ecuador.^26^ ^,^ ^42^ Since TcBat did not develop in several triatomine species, cimicids, vectors of *T. dionisii* in Europe, were considered potential transmitters of this genotype.^25^ Later, TcBat DNA was detected in natural infections of *T. sordida* in Brazil^43^ and *T. dispar* and *Cavernicola pilosa* sharing shelters with bats in Ecuador.^42^ Interestingly, despite the geographical ranges of TcBat, the identification of potential vectors, and description of its infectivity to mice,^25^ this lineage has not been reported in mammalian reservoirs. The analysis of more isolates, particularly from poorly studied wild reservoirs present in unexplored ecotopes, may elucidate this issue.

*Non-human primates and DTU TcIV* - A large study conducted by the group of Marta Teixeira in the Brazilian Amazonia concluded that TcI and TcIV circulate amongst non-human primates (NHP) and that these genotypes were transmitted by *Rhodnius* species in overlapping arboreal transmission cycles.^44^ Although sharing vectors and ecotopes, TcIV was not found in isolates from *D. marsupialis* captured in the same area, which were infected solely by TcI. Taken together, the data suggest that sympatric *T. cruzi* lineages of Amazonia circulate in independent transmission cycles determined by their preferential mammalian hosts.

TcI and TcIV DTUs in equal proportions were also reported infecting a colony of rhesus macaques in a NHP facility in central Texas (USA).^45^ Close to the facility, an active cycle of transmission was found in which raccoons harbored TcIV; opossums, TcI, and skunks both DTUs. No *T. cruzi* infection was observed in triatomines captured in the same region, maybe due to the limited sampling. TcI and TcIV are the prevalent DTUs in isolates of USA: TcIV in racoons (Carnivora) and lemurs (NHP) and, as expected, TcI in opossums. In the domestic cycle TcIV has been found in dogs (Carnivora).^46^

Contrasting observations were obtained in NHP species from southeastern Mexico, in which five DTUs (TcI, TcII, TcIII, TcV and TcVI) were identified.^47^ It should be noted the absence of TcIV, which in the examples mentioned above was abundant in NHPs from Brazil and USA. Coincidentally, the five DTUs identified in Mexican NHPs are predominant in human infections (see below).

*Golden lion tamarins and DTU TcII* - TcII infections are uncommon in the wild and abundant in the domestic transmission cycles in the southern and central regions of South America. One possible exception is the transmission cycle of TcII in the Atlantic Coastal Rainforest of Brazil where golden lion tamarins (NHP of the genus *Leontopithecus*) maintain stable TcII infection with long lasting high parasitaemia.^48^

*Unknown reservoirs of TcV and TcVI* - Except the description of TcV in some armadillos,^35^ wild reservoirs of TcV and TcVI are rare. On the other hand, these DTUs are prevalent in patients from Southern Cone countries (see below).

The body of evidence seems to indicate that there is a preferential association of *T. cruzi* DTUs to certain species / orders of mammals (Fig. 2). On the other hand, the factors that determine the adaptation of a given DTU to a reservoir and the exclusion of others are not known. Neither it is known whether the preferential association is determined solely by the genotypes of the species that interact or whether the adaptive phenotypic plasticity of the parasite plays a more important role. The elucidation of these aspects represents a fertile field for biological investigations and the answers will be of great contribution to the knowledge of host-parasite relationships.

*Triatomine vectors* - The vectors of *T. cruzi* are hematophagous insects of the subfamily Triatominae (Hemiptera: Reduviidae). All vector species were originally sylvatic and maintained an enzootic cycle with wild animals in variety of terrestrial or arboreal ecotopes. Anthropic environmental changes and damage to triatomine natural biotopes favored the domiciliation of some species that established domestic colonies, thus becoming vectors of ChD. The most important *T. cruzi* vectors are species of the genera *Triatoma*, *Rhodnius* and *Panstrongylus*.

Contrary to the scarcity of studies to unveil the elements involved in the preferential association of DTUs with wild reservoirs, knowledge of the DTUs-triatomine relationship is advancing.

The ability of *T. cruzi* strains to develop in different triatomine species was investigated mostly in *R. prolixus* and *T. infestans*, since laboratory colonies of these vectors are available. Some reports show that TcI strains complete their development in *R. prolixus*, unlike TcII strains that are eliminated.^49^ ^,^ ^50^ This is not the case for *T. infestans* that maintains both TcI and TcII genotypes administered in single or mixed infections.^51^ Taken together, these observations and others not reported due to space limitations suggest that *R. prolixus* would act as a biological filter selecting *T. cruzi* genotypes. The capacity of different vector species to select / eliminate the infecting strain *in vivo* would have epidemiological implications considering that, in nature, triatomines feed on different sources and can become infected with more than one *T. cruzi* strain.

The development of *T. cruzi* in the insect vector is a complex process whose success depends on the characteristics of both actors. The parasite must cope with digestive enzymes, innate immune response and the vector’s gut microbiota, whose characteristics vary between vector species (reviewed in^52^ ^,^ ^53^ ^,^ ^54^ ^,^ ^55^). In the digestive tract of the insect, the parasite undergoes a series of biochemical and morphological transformations that occur through the intimate association with vector’s structures. In the intestine, epimastigotes attach to the perimicrovillar membranes and actively divide. In the final portion of the digestive tract, epimastigotes attach to the rectal cuticle and differentiation into metacyclic trypomastigotes takes place (reviewed in^52^ ^,^ ^53^ ^,^ ^54^ ^,^ ^55^). *In vitro* studies have shown that the adhesion to these structures is mediated by components of the epimastigote surface such as glycoinositol phospholipids (GiPLs) and specific glycoproteins, whose molecular characteristics and abundance vary greatly between the evolutionary stages and *T. cruzi* genotypes^52^ ^,^ ^53^ (see below).

The more in-depth knowledge of the elements involved in *T. cruzi*-vector association will have implications for the design of strategies to interrupt parasite transmission.

**DTUs and human Chagas disease**

All DTUs are capable of promoting ChD. Yet, TcI, TcII, TcV and TcVI are the most prevalent DTUs in patients in Latin America.

We currently have a comprehensive view of the geographical distribution of DTUs in humans.^23^ ^,^ ^24^ ^,^ ^38^ ^,^ ^55^ ^,^ ^56^ In most studies, the characterisation of the infective DTU is done from the patient’s blood and, to a lesser extent, directly in the blood or tissue biopsies. In the first case, the parasites are obtained by haemoculture and amplified in liquid media. The process is time consuming and, in mixed infections, it can lead to the selection of particular parasite sub-populations. Another relevant drawback is the differential tissue tropism of the strains, which may determine that circulating parasites or parasites obtained from biopsies do not reflect the wide range of DTUs that infect the patient.^24^ ^,^ ^57^ Despite these limitations, the present consensus on the geographic distribution of DTUs prevalent in human chronic ChD is represented in Fig. 3 (data from^24^ ^,^ ^38^ ^,^ ^55^ ^,^ ^56^) and can be summarised as follows.

**Fig. 3:**
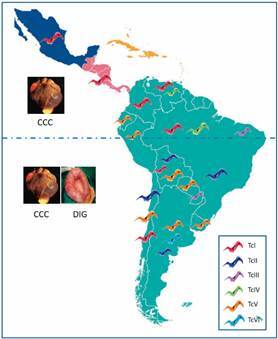
map shows prevalence of human *Trypanosoma cruzi* discrete typing units (DTUs) in Mexico and countries of Central and South America. The sizes of the parasite drawings reflect the relative occurrence of the DTUs in a given area (the larger, the more prevalent). The dashed line separates the geographical regions according to the prevalent chronic Chagas disease pathology: chronic Chagas cardiomyopathy (CCC) and digestive form (DIG).

TcI has a wide distribution and prevails in patients from Mexico, Central America, the Andean region and Amazonia. In central and southeastern Brazil TcII has high representation in Chagas infections. TcV is the most common DTU in Bolivia, Chile, Paraguay and Uruguay and is found in about 50% of patients in Argentina, who are also be infected with TcVI. Human infections by TcIV are rare, however this DTU is the secondary cause of ChD in Venezuela and reported in oral transmission outbreaks in the Brazilian Amazon. TcIII is mostly associated with sylvatic environment. For TcBat, there is no description in humans, except a report of TcBat DNA in mummies and in a child of Colombia.^29^ ^,^ ^30^

Initial evidence of the possible association between the parasite strain and the clinical manifestation came from the observation that isolates of patients from Venezuela, where megasyndromes are rare, are genetically different from isolates of patients from central and eastern Brazil, in which megasyndromes and / or cardiac manifestations are commonly observed.^58^ Characterisation of the parasites showed that Zymodeme Z1 (TcI in the present nomenclature^23^) was the main lineage in patients of Venezuela, whereas Z2 (TcII) was prevalent in Brazilian patients.^58^ ^,^ ^59^

Further studies employing various genotyping techniques confirmed the prevalence of TcII and TcV in individuals with a wide range of cardiac (CCC) and/or digestive (DIG) pathologies and TcI in patients with the indeterminate (IND) or CCC forms in northern Brazil, Colombia, Guatemala, Mexico and Panama (references in^24^ ^,^ ^38^ ^,^ ^56^). With these and other information, a hypothetical line could be traced on the map of Fig. 3 separating CCC and CCC + DIG manifestations. This representation indicates that DIG manifestation is absent (or rare) in Mexico, Central and northern South America as compared with other countries of South America. However, it should be pointed out that the information used to compile this picture is derived mostly from clinical reports rather than quantitative population-based epidemiological studies. Despite this caveat, two relevant questions, so far unanswered, can be raised: why does TcI not cause (or rarely cause) mega-syndromes? What characteristics of TcII and TcV would be responsible for the emergence of the DIG form?

Several research groups have investigated the potential association between *T. cruzi* genotype and chronic clinical outcome of ChD and some authors have performed a systematic review of the literature in search of evidence for this association.^24^ ^,^ ^38^ ^,^ ^57^ Despite the valuable information collected in these studies and recommendations for improving the study design, until now we have no clear answers.

On the other hand, growing evidence indicates that the clinical manifestation of ChD should result from the interplay between genetic characteristics of the infecting strain and characteristics of the human host, among which the immune response plays a pivotal role. It is out of the scope of this chapter to discuss the plethora of studies on immune responses during *T. cruzi* acute and chronic infections and their implication for the disease outcome. Nevertheless, it is worth mentioning that genetic variability of human genes associated to the immune system has been implicated in disease progression. Case-control studies revealed that polymorphisms in the hypervariable HLA I and II loci have been associated to chronic Chagas cardiomyopathy.^60^ ^,^ ^61^ In the same line, an association between a CD14 genotype and the development of Chagas dilated cardiomyopathy was established.^62^ On the other hand, the protective role of IL17A AA, IL18 AA, and IL1B TC genotypes against development/progression of cardiomyopathy has been reported.^63^

In addition to the involvement of polymorphisms of human immune genes, some structural components have also been associated to clinical ChD progression such as matrix metalloproteinases (MMP), known to be involved in extracellular matrix remodeling. While MMP-2 was proposed to be an early predictor for initial cardiac form, MMP-9 seems a potential marker of late fibrosis, where cardiac remodeling results in heart failure, apical aneurysm of the left ventricle, and hypertrophy.^64^ Recently, it has been shown that trans-sialidase neuraminidase activity of virulent strains from TcVI DTU is involved in MMP-2 activation, a phenomenon not detected for avirulent TcI strains investigated in that study. On the other hand, no significant difference was observed among the evaluated strains regarding the activation of MMP-9.^65^ Although these and other studies disclosed valuable prognosis predictors that may help the management of chagasic patients, we still do not know much about *T. cruzi* or DTU-specific molecules that favor the development of an immunological environment and/or structural changes associated with the different clinical outcomes of ChD.

**DTUs and genomic variability**

CL Brener, a member of the hybrid TcVI DTU, was the first *T. cruzi* genome sequenced. The genome draft, published in 2005 by El Sayed et al.,^66^ used Sanger-reads based assembly. Due to the hybrid nature of the organism and high repetitive content of the genome, the sequence assembly of CL Brener resulted in a fragmented genome. Later, the development of new sequencing technologies, such as short-Ilumina reads providing high-throughput and sequence accuracy, and long-PacBio and -Nanopore reads offering assembly continuity, has improved the quality of the assembled genomes. Recently, the genomes of a number of *T. cruzi* strains/DTUs such as, Brazil, Dm28c, Sylvio (TcI), Berenice and Y (TcII), Bug2148 (TcV), and TCC (TcVI) were assembled using long-reads or a combination of long- and short-reads.^67^ ^,^ ^68^ ^,^ ^69^ ^,^ ^70^ ^,^ ^71^ These improvements contributed to a better understanding of *T. cruzi* genome organisation and disclosed a more complete repertoire of gene content.

The scenario that emerged from these studies confirms that *T. cruzi* genome has a compartmentalised organisation named by Berná et al.^65^ as a core compartment containing conserved and hypothetical genes syntenic, i. e., displaying the same gene order, with *Leishmania* and *T. brucei* genomes; and a disruptive compartment comprising rapidly evolving multigene families, many of them encoding polymorphic surface antigens, such as Trans-sialidase (TS), mucin-associated surface proteins (MASP), *T. cruzi* mucins (TcMUC), and glycoprotein 63 (GP63), that play essential functions for parasite survival both in the insect vector and mammalian hosts (see below).

These surface gene families are dramatically expanded in the parasite genome, having 400-3,200 copies, that are clustered into several large haploid and heterogeneous arrays distributed along several chromosomes. In these clusters, although head-to-tail arrays of members of a given family were observed,^70^ ^,^ ^71^ in general, genes from the different families are alternated in a non-orderly fashion.^66^ ^,^ ^71^ ^,^ ^72^ It was speculated that this type of organisation may contribute for maintaining diversity of these gene families by avoiding sequence homogenisation through gene conversion.^72^ The disruptive compartment is also enriched with members of the retrotransposon hot spot (RHS) family and retroelements such as, VIPER, L1Tc, NARTc, DIRE, which may contribute to the generation of sequence variability through recombination.^70^ In summary, these studies reinforce the notion that distinct *T. cruzi* genomic regions are subjected to different selective pressures: while the core compartment exhibits low diversity, the disruptive compartment evolves under strong diversification pressure.

Comparative genomics within and among different *T. cruzi* DTUs reinforce the impressive diversity of genes encoding surface antigens. A comparative analysis of 35 TcI strains from different endemic regions in Latin America found a much higher density of single nucleotide polymorphisms (SNPs), that can promote amino acid changes, within surface multigene family clusters than in the core genome: 10 variants per kb in former compared to 0.4 variants per kb in the latter.^70^ Another study comparing the high-quality assembled genomes of the divergent Brazil (TcI) and Y (TcII) strains revealed no identical gene copies of TS, MASP and mucins families between the two genomes. On the contrary, genes encoded by the core compartment display almost no gene content difference and high level of sequence conservation between orthologs.^71^ Also, the copy number of the different gene families are quite distinct among strains and DTUs, ranging, for instance, from ~ 500 to 1,000 for mucin and ~ 1,500-3,200 for TS (Fig. 4), with hybrid strains tending to have the largest copy numbers.^67^ ^-^ ^73^ It is tempting to speculate that the distinct copy number and sequence variability of these multigene families between parental strains may have allowed that a number of members from both parental haplotypes might have been kept in the hybrid genomes after loss of redundant sequences.

**Fig. 4:**
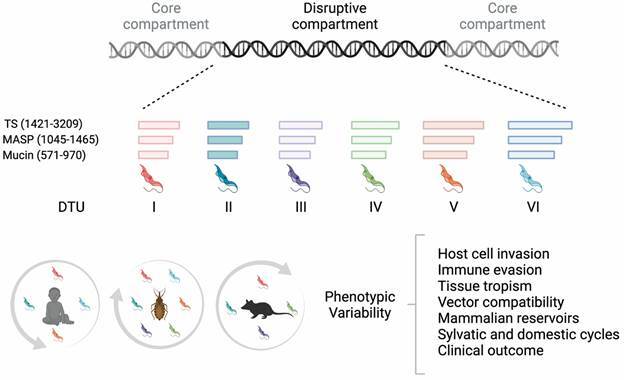
diversity of multigene families encoding surface proteins among *Trypanosoma cruzi* discrete typing units (DTUs) and parasite adaptability to different hosts and environments. *T. cruzi* genome is compartmentalised into the core and disruptive regions. The core compartment encodes conserved proteins mainly associated to house-keeping functions, while the disruptive compartment contains multicopy genes encoding polymorphic surface proteins, such as TS, MASP, and mucin, that display impressive intragenomic and inter-DTU sequence variability. These genes families are involved in several host-parasite interactions that may contribute to *T. cruzi* establishing the infection in different host cells and mammals and explore distinct niches. The coloured horizontal bars and number ranges represent the gene copy numbers of each family among strains of distinct DTUs. DTUs commonly associated to human (TcI, II, IV and V), vectors (all DTUs), and reservoirs (TcI, II, III and IV) infections are depicted in the circles at the bottom. Created with Biorender.com.

Many members of TS, MASP and mucin families are glycosylated and expressed at the surface of the circulating trypomastigotes via glycosylphosphatidylinositol (GPI) anchors, favoring a high dense packing of polymorphic proteins exposed at the parasite surface. Interestingly, it was demonstrated that members of TS and mucin families are not evenly distributed at the trypomastigote surface, but instead are organised into separate microdomains.^74^ ^,^ ^75^ Also, these proteins can be shed in the milieu via microvesicles.^74^ ^,^ ^75^ The astonishing expansion and variability of the genes encoding these surface proteins provide, therefore, a vast repertoire of distinct molecules exposed to the host. In fact, members of these families are important players in several host-parasite interaction mechanisms that allow parasite survive in the blood and mediate host cell invasion where it proliferates.

TS catalyses the transfer of sialic acid from glycoconjugates from the host to β-galactopyranose residues located in the parasite’s mucin proteins, generating a negatively charged surface that provides protection against the alternative complement pathway and antibody opsonisation.^76^ ^,^ ^77^ ^,^ ^78^ This family is also involved in processes of adhesion and invasion of epithelial,^79^ neural and glial cells.^80^ In the insect vector, proteins from this family expressed in the epimastigote form promote adhesion to endothelial cells in the triatomine’s intestine, as well as protect the parasite from the action of glycolytic enzymes.^78^

TcMUC mucins are the most abundant glycoproteins found at the surface of *T. cruzi*.^77^ Besides protecting the parasite from the immune response of both the vector and the mammalian host, mucins promote Ca^2+^ mobilisation in host cells, a process necessary for cell invasion.^81^ In epimastigotes, mucins play a role in the attachment of the parasite to the triatomine rectal ampoule, a critical step towards its differentiation into mammalian trypomastigote infective forms.^82^

MASP family was discovered recently during the sequencing of the CL Brener genome.^66^ MASP is characterised by conserved N- and C-terminal regions, that encode, respectively, for a signal peptide and a GPI anchor addition site, which flank a hypervariable central region exposed at the surface of trypomastigote forms.^72^ Evidence of differential MASP expression in TcI and TcVI strains and also during different parasite stages was also obtained.^83^ Experimental infections in murine model suggested that the pattern of expression of different MASP genes may vary in consecutive passages of *T. cruzi*, possibly playing a role in immune evasion during acute infection.^84^ Also, several peptides derived from MASP are recognised by sera from acutely infected mice and chronic chagasic patients.^84^ ^,^ ^85^ ^,^ ^86^

Several other functions were described for these families and are reported elsewhere.^87^ Nonetheless, as illustrated above, TS, MASP and mucins are involved in several parasite strategies that may allow *T. cruzi* establishing the infection in different mammalian and vector hosts and survive in distinct environments (Fig. 4). The availability of a couple of high-quality genomes unveiled a more complete picture of the variability of these families. It is important to emphasise, however, that these studies were based on the analyses of a limited number of assembled genomes, which in many cases were reconstructed as a haploid representation of the genome.^88^ Also, next-generation sequence analyses have revealed that many *T. cruzi* chromosomes are supra-numerary, i.e., have more than two copies, many of them containing surface antigens.^32^ Therefore, the complete repertoire of their sequences in *T. cruzi* taxon is still only partially disclosed. Nevertheless, the dramatic expansion and high level of polymorphism of these surface molecules in the different strains and DTUs may contribute to specific host-parasite/DTU interactions described in this review that has allowed the parasite to establish infection in different cell types and hosts and exploring several niches.
